# Investigation on optical, structural and electrical properties of solid-state polymer nanocomposites electrolyte incorporated with Ag nanoparticles

**DOI:** 10.1038/s41598-022-25304-0

**Published:** 2022-12-08

**Authors:** E. Salim, Wessam Hany, A. G. Elshahawy, A. H. Oraby

**Affiliations:** Physics Department, Faculty of Science, Mansoura University, Mansoura, Egypt

**Keywords:** Energy science and technology, Materials science, Physics

## Abstract

A solid polymer electrolyte based on polyvinyl alcohol (PVA)/carboxymethyl cellulose (CMC)/polyethylene 3,4-dioxythiophene: sodium polystyrene sulfonate (PEDOT:PSS) has been prepared with various concentrations of incorporated silver (Ag) nanoparticles (NPs) by using solution cast approach. The FTIR spectroscopic study revealed the complexation between the polymeric nanocomposite (PNC) and the Ag NPs. The X-ray diffraction (XRD) results infer that the semicrystalline phase of PNC decreases as the amount of incorporated Ag NPs increases. The transmission electron microscope (TEM) image revealed that Ag NPs have diameters ranging from 22 to 43 nm. Complex dielectric permittivity and alternating current (AC) electrical conductivity of nanocomposite films have been investigated in the frequency range from 0.1 Hz to 20 MHz at 30 °C. Dc conductivity (${\upsigma }_{\mathrm{dc}}$) values for the nanocomposite films are estimated from AC conductivity plots. The ${\upsigma }_{\mathrm{dc}}$ value was observed to increase from 1.98 × 10^−9^ to 2.29 × 10^−7^ S.cm^−1^ for the PNC system incorporated with optimal Ag NPs. From complex impedance (Z*) analysis, it has been found that the bulk electrical resistance (R_b_) of the PNC films decreases with increasing the Ag NPs content. Therefore, these obtained PNC films have promising applications in energy storage devices.

## Introduction

In recent years, the development of solid polymer electrolyte (SPE) has made great advances in sensors, high energy density batteries, electrochromic displays and windows, and photovoltaic cells due to its flexibility, electrochemical stability, long life, and safety^1,2^. Polyvinyl alcohol (PVA) has interesting characteristics and a wide range of uses. It possesses high dielectric strength, strong charge storage capacity, high elasticity, and good film formation via solution casting^3,4^. It also contains hydroxyl side groups, which can interact with a variety of substances via physical or chemical action^5^. Furthermore, Carboxymethyl cellulose (CMC), available as sodium salt NaCMC is a polyelectrolyte smart cellulose derivative, non-toxic, biodegradable, and has a large number of carboxyl groups (–COOH)^6^. So, it can create a strong connection with PVA via hydrogen bonding and subsequent cross-linking^7^. The addition of CMC to the PVA matrix can enhance the polymer’s properties^8^. To impart higher conductivity to the polymer blend, conductive additives with dispersibility and good conductivity are required. A large number of π-conjugated polymers can transport electrons, so the addition of polypyrrole^9–11^, polyaniline^12,13^, poly (3,4-ethyldioxythiophene) polystyrene sulfonate (PEDOT:PSS)^14,15^ into the polymer blend to enhance its conductivity. Among these polymers, PEDOT:PSS has high electrical conductivity ($\sigma$), excellent electrochemical stability, and film-forming capabilities. In addition, PEDOT:PSS has also been used as electrocatalysts^16^, capacitors^17,18^, transistors^19^, solar cells^20^, and sensors^21,22^. To produce nanocomposites, nanoparticles (NPs) within the polymeric blend interact and create molecular bridges. Large polymeric/nanofiller interfacial regions are formed as a result of the uniform distribution and excellent dispersion of these NPs, which improve the materials’ electrical and dielectric characteristics^23^. Recently, several experiments have been performed to manufacture nanocomposite materials incorporating a variety of inorganic NPs, including zinc oxide (ZnO)^24,25^, copper (Cu)^26^, gold (Au)^27^, and silver (Ag)^28^, with different polymers. Among these inorganic NPs, Ag has the highest electrical conductivity ($\upsigma \hspace{0.17em}\hspace{0.17em}=\hspace{0.17em}$6.3×${10}^{5}$ S.cm^−1^ at 20 °C) of any metal and is relatively inexpensive compared to other noble metals^29^.

In this work, SPE-based PNC films, PVA/CMC/PEDOT:PSS/various weights Ag NPs, were prepared. XRD, FTIR, and TEM were used to examine the structural properties of the produced nanocomposite films. Moreover, the impact of the Ag NPs content on the optical, electrical and dielectric properties of the prepared films has been investigated.

## Experimental details

### Materials

Polyvinyl alcohol (PVA, M.W. = 89,000–98,000, 99+% hydrolyzed), Poly(2,3-dihydrothieno-1,4-dioxin)-poly(styrene sulfonate) (PEDOT:PSS, 1.3 wt% dispersion in H_2_O), and carboxymethylcellulose (CMC, average M.W. = 250,000) were purchased from Sigma-Aldrich. Ag NPs (M.W. = 107.87) were purchased from Nanjing Chemical Reagent Co. Ltd. All the chemicals were analytically pure and used without purification.

### Preparation of PVA/CMC/PEDOT: PSS/Ag NPs

Firstly, 1 g PVA and 0.5 g CMC were dissolved in 50 ml deionized water (DW) at 90 °C and 70 °C respectively, and then the two solutions were mixed and stirred for 3 h until completely dissolved. Secondly, five solutions of 10 ml of the above dissolved PVA/CMC and 0.3 ml of PEDOT:PSS in H_2_O were taken and mixed under stirring for 1h, and then 0, 1, 3, 5, 7 mg Ag NPs dispersed in 0.5 ml DW were added and continued to stir for 1 h, respectively. Finally, the resulting solutions were poured into polystyrene Petri dishes and evaporated at 50 °C. The as-prepared films with a thickness of 10–20 microns were labeled PCPP0, PCPP1, PCPP2, PCPP3, and PCPP4 based on the amount of Ag NPs added gradually.

### Characterization

X-ray diffraction (XRD) patterns of the nanocomposite films were recorded at room temperature using DIANO corporation USA with CuKα radiation in the Bragg angle 2θ = 5°–70°. Fourier transform infrared (FTIR 430-JASCO, Japan) spectrometer measurements were recorded within the 400–4000 cm^−1^ range. The optical absorptions of the films were obtained using a UV–visible spectrophotometer (JASCO V-630-Japan) at λ = 190–900 nm. The size of Ag NPs was investigated by transmission electron microscope (TEM, JEOL/ JEM/1011, Japan). The scanning electron microscope (SEM, JEOL-JSM 6510/LV/250, U.S.A., at magnification 6,000X) was used to investigate the surface topography. Impedance and dielectric characteristics of the PNC films were carried out in the 10^−1^ Hz–10 MHz range at room temperature (305 K) using broadband dielectric spectroscopy (Novo control Turnkey Concept 40 System).

## Results and discussion

### FT-IR analysis

**Figure 1:**
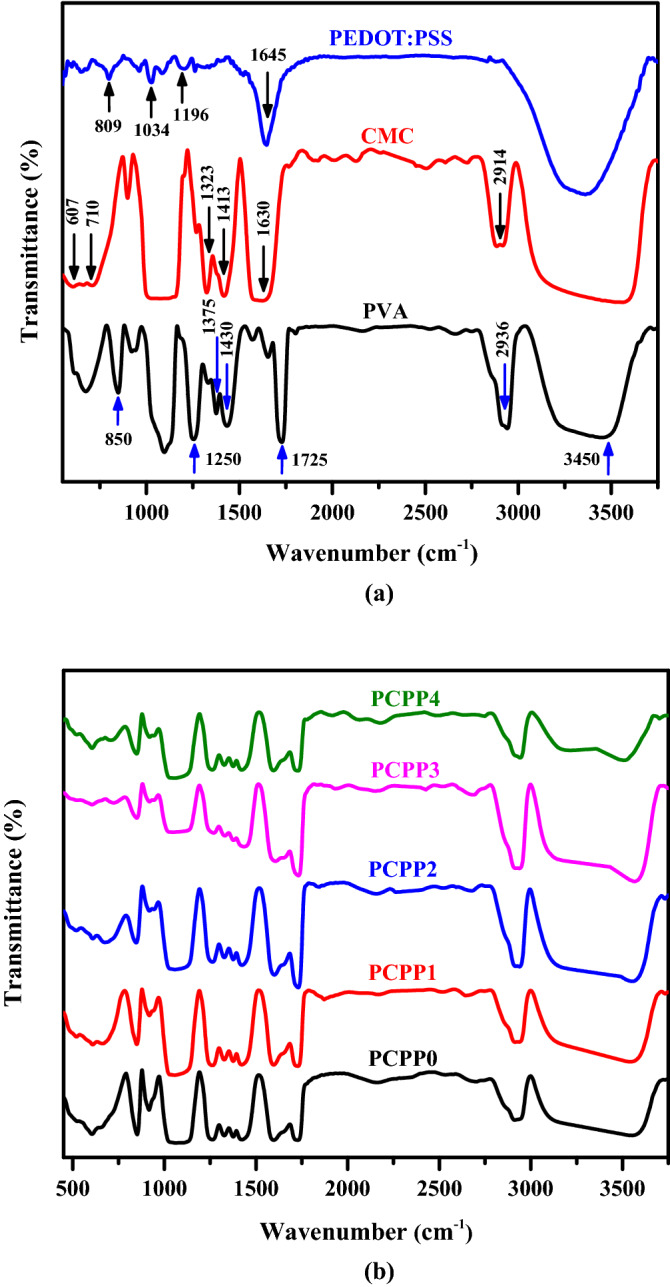
(**a**) FTIR spectra of PVA, CMC, PEDOT: PSS, and (**b**) PCPP with various Ag NPs weights films.

Fourier-transformed infrared (FTIR) spectroscopy was used to investigate the chemical structure of solid films. Figure 1a shows the FTIR spectra of PVA, CMC, and PEDOT:PSS. According to the FTIR spectrum of pure PVA, there is a wide characteristic peak observed at 3450 cm^−1^ assigned to the stretching vibration of intermolecular and intramolecular –OH bonds. The peaks at 2936 and 1725 cm^−1^ pertain to the stretching vibration of C–H and carbonyl C**=**O groups, respectively^30,31^. Also, the noticed peaks at 1430 and 1375 cm^−1^ are assigned to the bending and wagging of CH_2_ vibrations, respectively^32^. However, peaks at 1250, and 850 cm^−1^ are due to the stretching vibration of C–O and C–C groups, respectively. The above-all peaks confirm the existence of PVA. The peaks at 2914, 1630, 1413, 1323, 710, and 607 cm^−1^ are all correlated to pure CMC^33^. For PEDOT:PSS, the peak around 3400 cm^−1^ is due to the O–H stretching, the peak at 1645 cm^−1^ is due to the bending of the C=C group, peaks at 1196 and 1034 cm^−1^ are due to the stretching of C–O–C bond, and peak at 809 cm^−1^ is attributed to C–S bonds in thiophene backbone^34,35^. The characteristic peaks of PVA, CMC, and PEDOT:PSS can be noticed from the PVA/CMC/PEDOT:PSS (PCPP0) nanocomposite film, and the results of various weights of incorporated Ag NPs into PCPP in Fig1b. The oxygen-containing functional group (SO_3_ –H^+^) of PEDOT:PSS can interact and cross-link with PVA/CMC via hydrogen bonding^36,37^. With the following Ag NPs incorporating process into PCPP, the broadening and decreasing in the peak intensity suggest the physical interaction occurred between them^38^.

### X-ray diffraction (XRD) analysis

**Figure 2:**
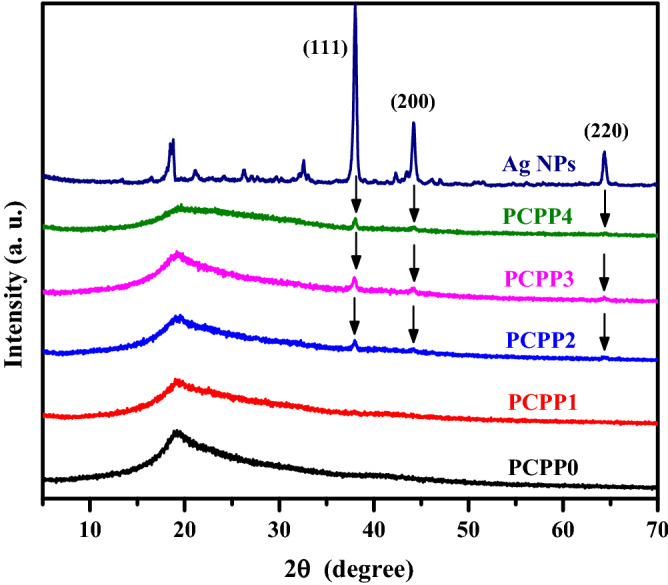
XRD spectra of PCMPP with various Ag NPs concentrations films.

Figure2 shows the XRD patterns for PCPP0, PCPP1, PCPP2, PCPP3, and PCPP4 from 5° to 70°, confirming the successful complexation between PVA, CMC, and PEDOT:PSS. It have been reported that PVA, CMC, and PEDOT:PSS have diffraction peaks lying at 2θ = 19.5°^39^, 2θ = 20.7°^40^ and 2θ = 25.6°^41^, respectively. It can be observed that the PCPP0 shows the existence of a semicrystalline phase with the diffraction peak centered at 2θ = 19.3°. This peak becomes more broadening and less intense as the Ag NPs increase which suggests the transference of PCPP from semicrystalline to amorphous structure. In addition, the higher weights of Ag NPs showed diffraction peaks at 2θ = 38°, 2θ = 44°, and 2θ = 64°, corresponding to (111), (200), and (220) lattice planes, respectively^42^.

## Optical absorption study

**Figure 3:**
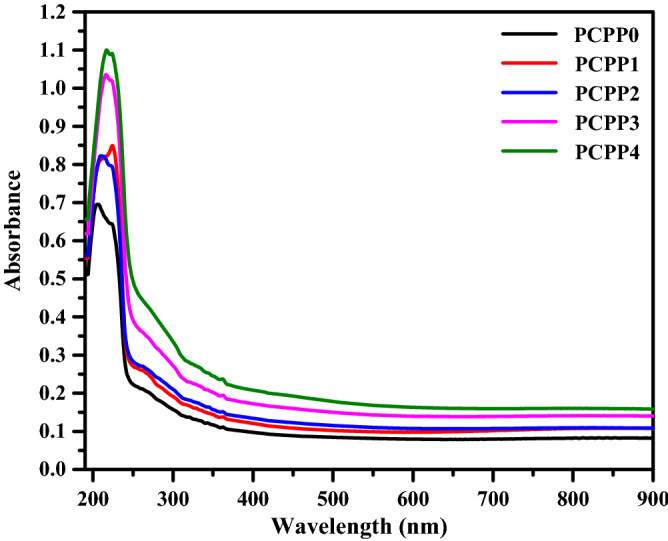
UV–vis absorption spectra of PVA/CMC/PEDOT: PSS with various weights of Ag NPs.

**Figure 4:**
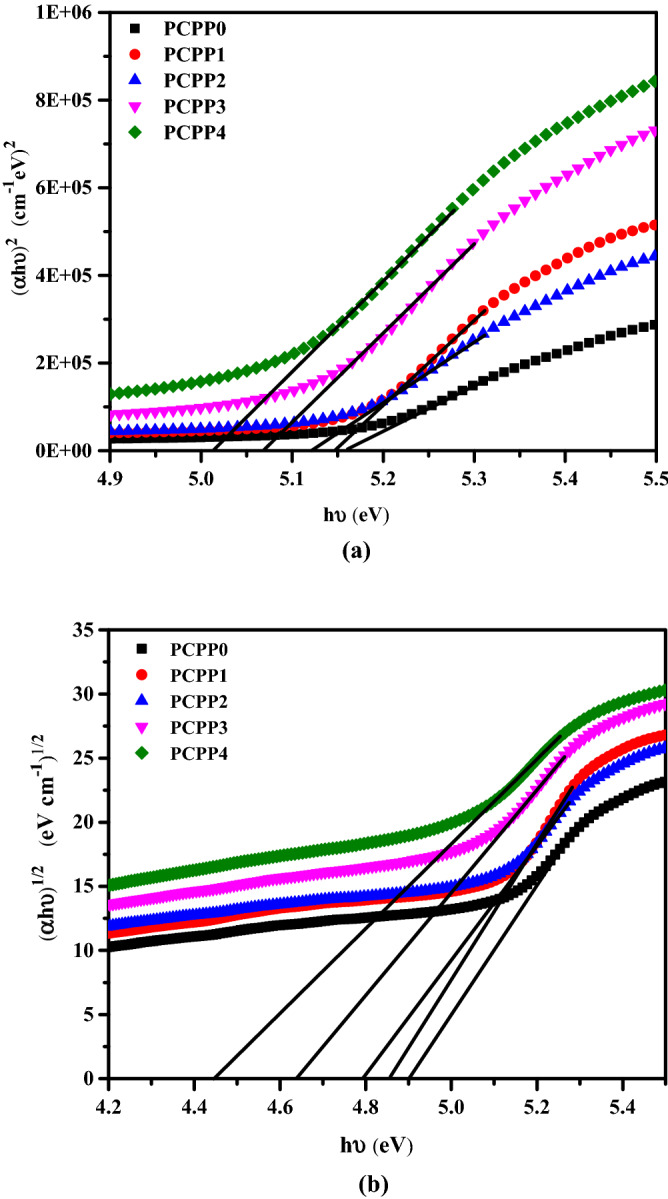
(**a**) (αhυ)^2^ and (**b**) (αhυ)^1/2^ versus hυ of PVA/CMC/PEDOT: PSS with various weights of Ag NPs.

The optical characteristics of the nanocomposite films (PCPP0 → 4) are investigated using a UV–vis spectrophotometer in the wavelength range 190–900 nm as shown in Fig. 3. An absorption band and hump are observed for all films at about 205 and 265 nm, respectively, which are ascribed to π-π* transition^43^. It is seen that there is an increase in the absorption peaks and is red-shifted with the increase of the incorporated Ag NPs. The optical energy bandgap (E_g_) of the prepared films could be obtained from the absorption spectra using Tauc’s formula^44^:

1 $$\upalpha {\text{h}}\upupsilon =\upbeta \left( {{\text{h}}\upupsilon - {\text{E}}_{{\text{g}}}}\right)^{{\text{m}}}$$

where α is the absorption coefficient, β is a constant, and m is an empirical index of the transition modes. To calculate the direct optical energy gap (${\mathrm{E}}_{\mathrm{g}}^{\mathrm{d}}$) for the investigated films, the quantity (αhυ)^2^ is plotted as a function of photon energy (hυ) (Fig. 4a). For undoped film PCPP0, the optical energy gap was observed to be 5.16 eV while for incorporated films PCPP1 → 4, the values were 5.15, 5.12, 5.07, and 5.01 eV, respectively. On the other hand, the indirect optical energy gap (${\mathrm{E}}_{\mathrm{g}}^{\mathrm{in}}$) were calculated using (αhυ)^1/2^ versus (hυ) plots (Fig. 4b) and found to be 4.9 eV for PCPP0 and 4.85, 4.79, 4.64, and 4.44 eV for dopped films, respectively. The values of direct and indirect optical energy gaps are listed in Table 1. It is clear that the decrease in the values of ${\mathrm{E}}_{\mathrm{g}}^{\mathrm{d}}$ and ${\mathrm{E}}_{\mathrm{g}}^{\mathrm{in}}$ on doping PCPP with Ag NPs may be attributed to the formation of charge-transfer complexes^45^. In addition, the enhancement in the absorption coefficient of the PNC films may be related to the agglomeration of Ag NPs, which may scatter the light within the PNC samples^46^.

**Table 1:** The extracted values of direct and indirect energy bandgap for PVA/CMC/PEDOT: PSS with various weights of Ag NPs.

Films	${\mathrm{E}}_{\mathrm{g}}^{\mathrm{d}}$(eV)	${\mathrm{E}}_{\mathrm{g}}^{in}$(eV)
PCPP0	5.16	4.9
PCPP1	5.15	4.85
PCPP2	5.12	4.79
PCPP3	5.07	4.64
PCPP4	5.01	4.44

### Transmission electron microscope (TEM)

**Figure 5:**
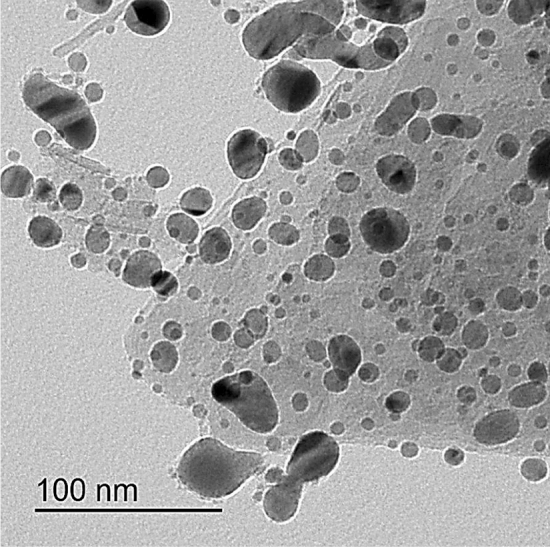
TEM images of Ag NPs.

Figure 5 shows the TEM image of Ag NPs. It has a nearly spherical shape with sizes ranging from 22 to 43 nm.

### Morphological studies

**Figure 6:**
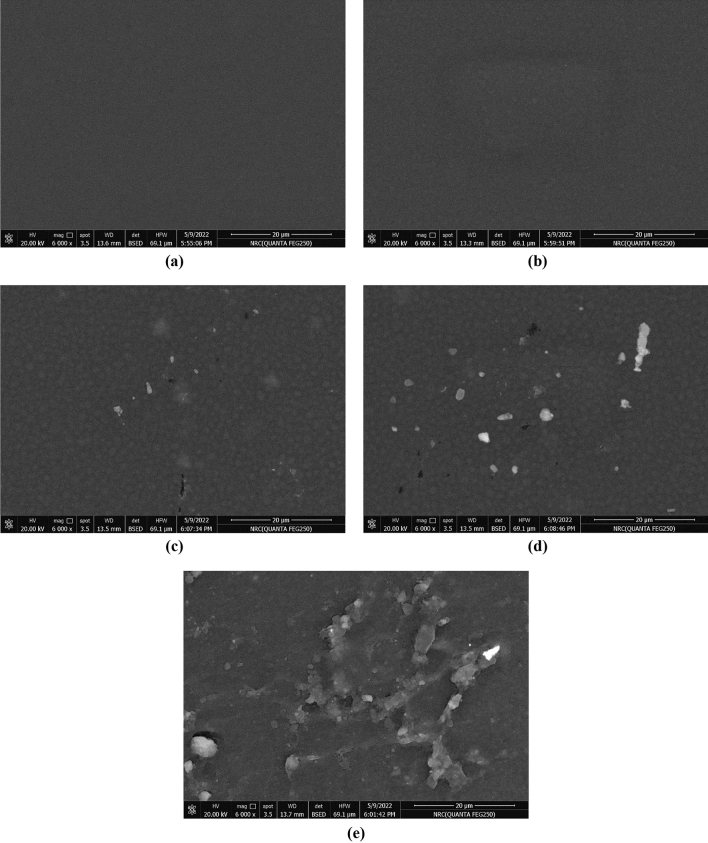
SEM images of (**a**) PCPP0, (**b**) PCPP1, (**c**) PCPP2, (**d**) PCPP3, and (**e**) PCPP4 at magnification 6,000 times.

Scanning electron microscope (SEM) is a commonly used technique for identifying the formation and growth of agglomerated metallic NPs that leak to the surface of polymer nanocomposites^47,48^. Figure 6a–e show the surface morphology of PCPP films. From the top view of the PCPP0 nanocomposite film, the micrograph appeared to be smooth and promoted the good quality of the prepared films. When the Ag NPs content increased gradually, aggregated particles were formed, and their sizes increased. The consequences of the morphological alterations were revealed in the dielectric characteristics of the nanocomposite films and are described further below.

### AC conductivity

**Figure 7:**
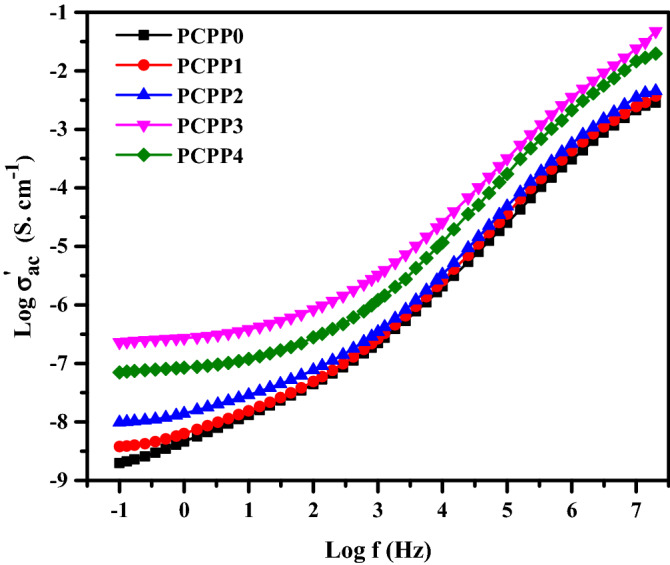
Log AC conductivity versus log frequency for all films at room temperature.

Figure 7 shows the plot of AC conductivity as a function of frequency in the range of 0.1 Hz–20 MHz at room temperature. As noted, the behavior of ${\upsigma }_{\mathrm{ac}}^{\mathrm{^{\prime}}}$ increases nonlinearly with increasing frequency. This can be attributed to the hopping mechanism of charge carriers that acquires by providing the electrical field which in turn increases the relaxation frequency and so enhances the conductivity^49^. It is found that the frequency-dependent spectra of the nanocomposite films exhibit three distinguished regions; (i) the low-frequency dispersion region, which is related to the accumulation of charge (electrode polarization) at the electrode/polymer interface, (ii) the mid-frequency independent plateau region, which is attributed to DC conductivity ${\upsigma }_{\mathrm{dc}}$, and (iii) high-frequency dispersion region which is attributed to short-range ion transport, which is caused by the charge carrier’s Coulomb interaction. So, the ${\upsigma }_{\mathrm{dc}}$ values of the films can be estimated using Jonscher’s power law^50,51^;

2 $$\upsigma _{{{\text{ac}}}}^{^{\prime}} \left(\upomega \right) = \upsigma _{{{\text{dc}}}} + {\text{A}} \upomega ^{{\text{m}}}$$

where A represents the frequency-independent pre-exponential constant, $\upomega$ is the angular frequency ($\upomega \hspace{0.17em}$= 2 $\mathrm{\pi f}$), and m is the power-law exponent (0 ˂ m ˂ 1). The ${\upsigma }_{\mathrm{dc}}$ of the undoped and doped Ag NPs films has been calculated by intersecting extrapolated plateau region of ${\upsigma }_{\mathrm{ac}}^{\mathrm{^{\prime}}}$- axis. It is noticed that the ${\upsigma }_{\mathrm{dc}}$ value increased from 1.98 × 10^−9^ to 7.41 × 10^−8^ S.cm^−1^ for PCPP0 and PCPP3 films, respectively. The increase in ${\upsigma }_{\mathrm{dc}}$ values of the nanocomposite films with the increase of dispersed Ag NPs contents indicates that there is either increase in charge carrier mobility or an increase in Ag ion carriers that contribute to the conductivity and polarization mechanisms. While the higher the content of Ag NPs in the PCPP4 film, the lower the ${\upsigma }_{\mathrm{dc}}$ value. This decrease may be attributed to the reduced Ag NPs which might act as grain boundaries and impede the Ag ion carriers to be transported through the polymeric matrices. Therefore, a few Ag ions participate in the conductivity and polarization.

### Dielectric studies

**Figure 8:**
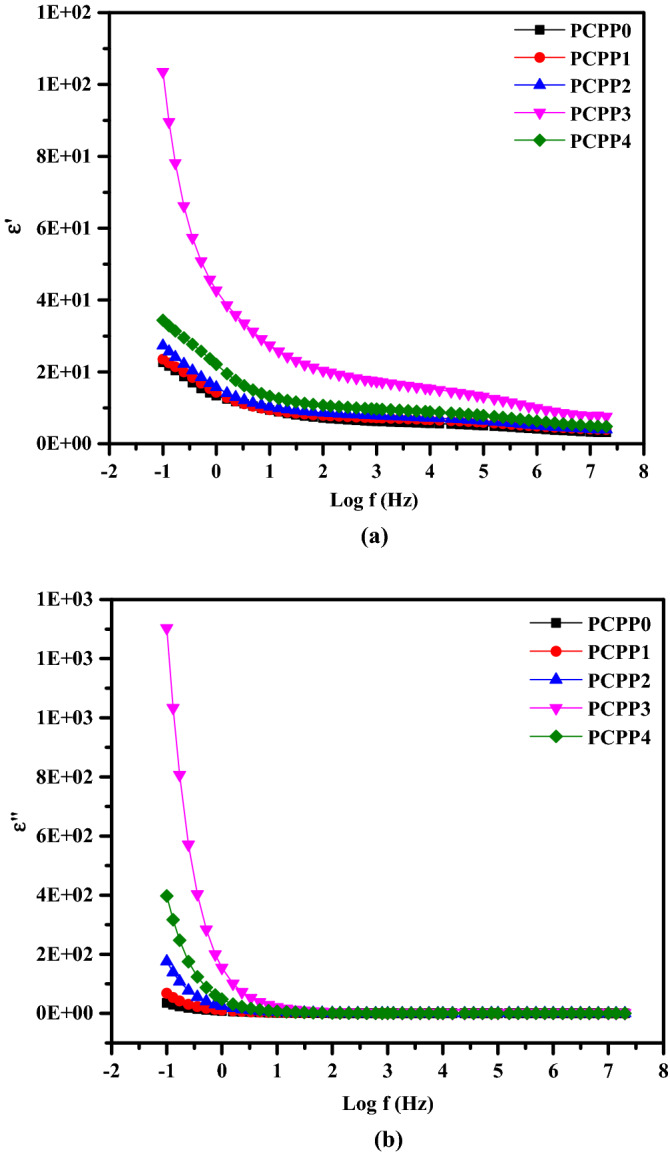
Frequency-dependent (**a**) real part ε′ and (**b**) imaginary part ε″ of the complex dielectric permittivity for all films at room temperature.

It is well known that the dielectric spectroscopy of polymeric materials is a powerful technique to understand the variation of electrical conductivity mechanisms. So, the dielectric data of the nanocomposite films were analyzed using the complex dielectric constant ${\upvarepsilon }^{*}$ as following^52,53^:

3 $$\upvarepsilon ^{*} = {\upvarepsilon} {^\prime } - {\text{j}} {\upvarepsilon} {^\prime } {^\prime } ,\; {\upvarepsilon} {^\prime } = \frac{{{\text{Cd}}}}{{{\text{A}} {\upvarepsilon} _{{\text{o}}} }}, \;{\varepsilon} ^{\prime \prime } = \frac{{\upsigma _{{{\text{ac}}}} }}{{\upvarepsilon _{{\text{o}}}\upomega }}$$

where ${\upvarepsilon }^{\mathrm{^{\prime}}}$ is the real part of the relative permittivity (dielectric constant), ${\upvarepsilon }^{\mathrm{^{\prime}}\mathrm{^{\prime}}}$ is the imaginary part of the relative permittivity (dielectric loss), C is the capacitance of the film, d is the film’s thickness, A is the surface area of the electrodes, and ${\upvarepsilon }_{\mathrm{o}}=$ 8.85 10^−12^ F/m is the free space-permittivity. The ${\upvarepsilon }^{\mathrm{^{\prime}}}$ value measures the amount of charge that can be stored by the material, whereas the ${\upvarepsilon }^{\mathrm{^{\prime}}\mathrm{^{\prime}}}$ the amount of energy lost. Plots of both ${\upvarepsilon }^{\mathrm{^{\prime}}}$ and ${\upvarepsilon }^{\mathrm{^{\prime}}\mathrm{^{\prime}}}$ against log f of the nanocomposite films at room temperature are shown in Fig. 8a and b, respectively. It is seen that the behavior of ${\upvarepsilon }^{\mathrm{^{\prime}}}$ and ${\upvarepsilon }^{\mathrm{^{\prime}}\mathrm{^{\prime}}}$ increase non-linearly with the decrease in frequency at room temperature. This phenomenon is more noticeable at lower frequencies (f ˂ 10^2^ Hz). The increase in permittivity with the decrease of frequency indicates that the system shows interfacial polarization at a low frequency^54^. On the other hand, the response of charge carriers at low frequency is faster with an externally applied electric signal, resulting in a higher value of ${\upvarepsilon }^{\mathrm{^{\prime}}}$. The values of ${\upvarepsilon }^{\mathrm{^{\prime}}}$ and ${\upvarepsilon }^{\mathrm{^{\prime}}\mathrm{^{\prime}}}$ increased with increasing Ag NPs content until PCPP3 nanocomposite film. This can be attributed to an increase in the density of Ag ion carriers as well as an increase in polarization. However, when the Ag NPs increased above the PCPP3 sample, the values of the dielectric constant and dielectric loss were reversed. This decrease in ${\varepsilon }^{\mathrm{^{\prime}}}$ and ${\varepsilon }^{\mathrm{^{\prime}}\mathrm{^{\prime}}}$ values could be related to the reduction of Ag-ion carriers to Ag particles. At high frequency, the permittivity values are found almost independent of the frequency due to the charge carriers being unable to reorient themselves with the applied electric field. Furthermore, ion oscillations may be the only source of the dielectric constant at high frequency, so ${\varepsilon }^{\mathrm{^{\prime}}\mathrm{^{\prime}}}$ is frequency independent. These results suggest that the dielectric constant might be used to evaluate the conductivity of the nanocomposite films.

### Impedance analysis

**Figure 9:**
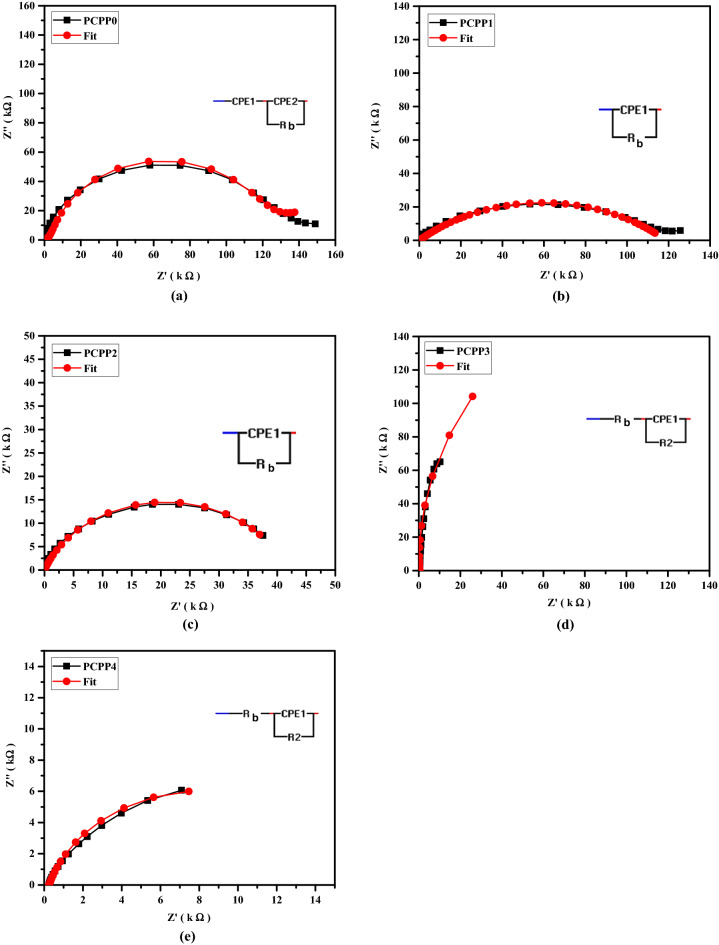
(**a**–**e**) Nyquist plots of the prepared nanocomposite films.

Impedance spectroscopy is a well-known technique used to determine ion dynamics in polymer electrolyte systems. In the current SPE, ac impedance measurements were used to illustrate electrolyte conductivity and frequency-dependent action. The Cole–Cole plots (Nyquist plots) for nanocomposite films based on SPE at room temperature are shown in Fig. 9. The obtained complex impedance spectra show two distinct regions; the high-frequency semi-circle region, which is due to the ionic conducting nature in the bulk of the polymer electrolytes, and the low-frequency spike, which is due to the blocking electrode (space charge polarization effect)^55^. To clarify the relationship between microstructure and electrical characteristics, impedance data is often represented as an equivalent electric circuit consisting of resistance and capacitance. The impedance data were fitted with an equivalent circuit using EIS software. As shown in the inset Fig. 9a–e, this equivalent circuit of the PNC films consists of a parallel combination of resistance R_b_ and fractal capacity CPE1 in series with other fractal capacity CPE2. A CPE is a constant phase element that indicates a deviation from the ideal Debye-type model. R_b_ represents the bulk resistance in this model, and it can be calculated using the low-frequency intercept of the semi-circle on the Z′ axis. CPE1 and CPE2 are simple distributed components that provide impedance with a constant phase angle in the complex plane. The impedance of the CPE is described by the following formula:

4 $${\text{ZCPE}} = \frac{1}{{{\text{Q}} \left( {{\text{i}}\upomega } \right)^{{\text{n}}} }}$$

where Q represents the numerical value of 1/$\left|Z\right|$ at $\upomega \hspace{0.17em}$= 1 rad s^−1^ and n represents the phase of the components, which gives the degree of deviation from the pure capacitor. The parameters of the equivalent electric circuit model are derived by fitting the curves in Fig. 9 are listed in Table 2. It can be noticed that the R_b_ decreases as the Ag NPs concentration increases. Also, the semi-circle centers are observed to be below the Z′- axis. This implies that the ions’ relaxation in the prepared samples is non-Debye kind. The lack of a high-frequency semi-circle indicates that overall conductivity is mostly due to ion conduction^56,57^.

**Table 2:** The extracted parameters of the equivalent electric circuit model.

Films	Fitting parameters				
	R_b_ (k Ω)	Q_1_ (F)	n_1_	Q_2_ (F)	n_2_
PCPP0	142	3.3 × 10^−5^	0.3	4.18 × 10^−7^	0.94
PCPP1	118	6.35 × 10^−7^	0.46		
PCPP2	41	8.14 × 10^−6^	0.77		
PCPP3	0.450	2.48 × 10^−5^	0.99		
PCPP4	0.252	6.61 × 10^−5^	0.82		

## Conclusions

In summary, we incorporated various amounts of Ag NPs into PVA/CMC/PEDOT:PSS composite to prepare the solid nanocomposite electrolyte. The solution casting approach was used to prepare the nanocomposite films, and the obtained films were physically and electrically characterized. PVA and CMC are utilized as hydrophilic structures, which provide flexibility to the films. The presence of the PEDOT:PSS provides the conductivity of the films. FT-IR spectra reveal the complex formation between the Ag NPs and PVA/CMC/PEDOT: PSS nanocomposites, and the complexation of polymers composite each other. The XRD pattern shows that the amorphous nature of the films increases with the addition of the Ag NPs, and three characteristic diffraction peaks confirmed the presence of Ag NPs. Furthermore, it was noticed that increasing the Ag NPs concentration has a significant influence on reducing the optical band gap energies. SEM images revealed the miscibility between the PVA, CMC, and PEDOT:PSS by a homogeneous smooth surface. Moreover, the surface structure of the PNC films is influenced by the Ag NPs filling ratio. The optical characteristics of the PNC films indicated that as the concentration of the Ag NPs increased, the absorbance increased while the optical band gap decreased. The AC conductivity values for the prepared films were increased up to the optimal nanocomposite film PCPP3, which can be ascribed to the increased contribution of Ag ion carriers to the conductivity and polarization mechanisms. Both ${\upvarepsilon }^{\mathrm{^{\prime}}}$ and ${\upvarepsilon }^{\mathrm{^{\prime}}\mathrm{^{\prime}}}$ also increased at the optimal nanocomposite film PCPP3 which in turn facilitates the charge carriers’ mobility. Also, the reduction in the diameter of semi-circle impedance plots indicates an increase in ionic conductivity. Further addition of Ag NPs in the composite films reduced the ${\upsigma }_{\mathrm{dc}}$, ${\upvarepsilon }^{\mathrm{^{\prime}}}$, and ${\upvarepsilon }^{\mathrm{^{\prime}}\mathrm{^{\prime}}}$ due to the increase in the rate of Ag ion reduction.

## Data Availability

All data generated during this study are included in this published article.
